# Diethyl 1-(4-methyl­phen­yl)-3-phenyl-5-oxopyrrolidine-2,2-dicarboxyl­ate

**DOI:** 10.1107/S1600536810028552

**Published:** 2010-07-24

**Authors:** Jayanta Kumar Ray, Gopa Barman, M. Canle L., M. I. Fernández P., J. A. Santaballa

**Affiliations:** a Department of Chemistry, Indian Institute of Technology, Kharagpur 721 302, India; b Departamento de Química Física e Enxeñería Química I, Facultade de Ciencias, Universidade da Coruña, Rúa Alejandro de la Sota 1, E-15008 A Coruña, Spain

## Abstract

In the title compound, C_23_H_25_NO_5_, the lactam ring adopts an envelope conformation and both eth­oxy­carbonyl side chains show an *s-cis* conformation: one is nearly planar, the dihedral angle between CO_2_ and OCH_2_CH_3_ groups being 7.95 (14)° and the other is almost orthogonal, the C—O—C—C torsion angle being 85.33 (9)°. Dimers related by inversion symmetry are stabilized by C—H⋯O hydrogen bonds. The crystal structure is consolidated by weak intermolecular C—H⋯O inter­actions. Weak intra­molecular inter­actions of the same kind also occur.

## Related literature

The title compound may show anti­bacterial activity as has been found in other γ-lactam derivatives. For related structures see: Nigam *et al.* (1989 ▶); Ray *et al.* (1994 ▶, 1998 ▶, 2004 ▶, 2010 ▶); Kandasamy *et al.* (1995 ▶). For conformational analysis, see: Cremer & Pople (1975 ▶); Rao *et al.* (1981 ▶). For hydrogen bonding, see: Desiraju (2005 ▶).
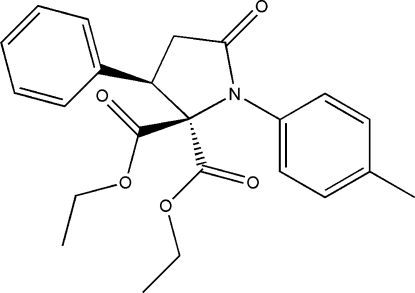


## Experimental

### 

#### Crystal data

C_23_H_25_NO_5_
*M* *_r_* = 395.44
Triclinic, 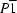

*a* = 9.4905 (2) Å
*b* = 10.6167 (2) Å
*c* = 10.8198 (2) Å
α = 93.014 (1)°
β = 95.167 (1)°
γ = 110.537 (1)°
*V* = 1012.60 (3) Å^3^
*Z* = 2
Mo *K*α radiation
μ = 0.09 mm^−1^
*T* = 100 K
0.42 × 0.30 × 0.12 mm

#### Data collection

Bruker APEXII area-detector diffractometer
Absorption correction: multi-scan (*SADABS*; Bruker, 2009 ▶) *T*_min_ = 0.964, *T*_max_ = 0.996
14991 measured reflections
3672 independent reflections
3331 reflections with *I* > 2σ(*I*)
*R*_int_ = 0.020

#### Refinement

*R*[*F* ^2^ > 2σ(*F* ^2^)] = 0.031
*wR*(*F* ^2^) = 0.091
*S* = 1.87
3672 reflections
362 parameters
All H-atom parameters refined
Δρ_max_ = 0.22 e Å^−3^
Δρ_min_ = −0.22 e Å^−3^

### 

Data collection: *APEX2* (Bruker, 2009 ▶); cell refinement: *SAINT* (Bruker, 2009 ▶); data reduction: *SAINT*; program(s) used to solve structure: *SHELXS97* (Sheldrick, 2008 ▶); program(s) used to refine structure: *SHELXL97* (Sheldrick, 2008 ▶); molecular graphics: *ORTEP-3* (Farrugia, 1997 ▶) and *PLATON* (Spek, 2009 ▶); software used to prepare material for publication: *SHELXL97*.

## Supplementary Material

Crystal structure: contains datablocks global, I. DOI: 10.1107/S1600536810028552/rn2065sup1.cif

Structure factors: contains datablocks I. DOI: 10.1107/S1600536810028552/rn2065Isup2.hkl

Additional supplementary materials:  crystallographic information; 3D view; checkCIF report

## Figures and Tables

**Table 1:** Hydrogen-bond geometry (Å, °)

*D*—H⋯*A*	*D*—H	H⋯*A*	*D*⋯*A*	*D*—H⋯*A*
C2—H2*A*⋯O2^i^	0.985 (14)	2.529 (14)	3.5096 (14)	173.4 (10)
C3—H3⋯O4	0.984 (13)	2.369 (13)	2.8814 (14)	111.7 (9)
C6—H6⋯O2	0.955 (15)	2.573 (14)	3.3143 (14)	134.7 (11)
C13—H13⋯O2^i^	0.975 (14)	2.453 (14)	3.4128 (14)	168.3 (12)
C15—H15⋯O1^ii^	0.987 (15)	2.462 (15)	3.2184 (15)	133.2 (10)
C22—H22*A*⋯O1^iii^	0.963 (13)	2.513 (14)	3.2100 (15)	129.2 (9)
C22—H22*B*⋯O4^iv^	0.983 (13)	2.579 (13)	3.2426 (14)	124.9 (10)

Symmetry codes: (i) 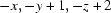; (ii) 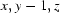; (iii) 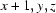; (iv) 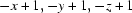.

**Fig. 1.**
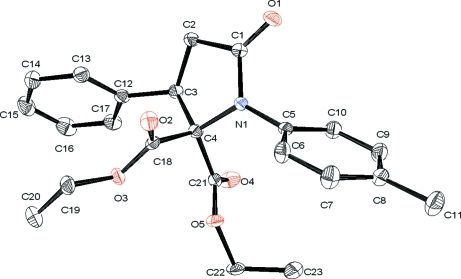
View of the title compound showing the atomic numbering and 50% probability displacement ellipsoids. H atoms are not shown for clarity.

## supplementary crystallographic information

### Comment

In addition to the γ-lactam unit (N1/C1—C4), the title compound contains two phenyl rings (C5—C10) and (C12—C17), and two ethoxycarbonyl side chains, with bond distances and angles within typical values. The asymmetric unit of the title compound with our numbering scheme is in Figure 1. The γ-lactam unit (N1/C1—C4) adopts an envelope conformation. Atom C3 deviates 0.4804 (11)Å from the mean plane passing through the remaining atoms in the ring (r.m.s. 0.026 Å). In the envelope the atom C3 is the flap, with puckering parameters q2 = 0.3030 (12)Å and φ_2_ = 291.9 (2)° (Cremer & Pople, 1975), and a pseudo-rotation angle P = 90.0 (1)°, and a maximum torsion angle τ_m_ = 30.6 (1)° (Rao *et al.*, 1981) when the bond reference is N1—C1. The planar portion of the γ-lactam unit (N1—C1—C2—C4) forms dihedral angles of 69.53 (7)° and 67.61 (7)° with rings (C5—C10) and (C12—C17) respectively, and the dihedral angle between them is 56.66 (6)°. The ethoxycarbonyl side chain involving O2—C18—O3—C19—C20 adopts a s-*cis* conformation, with atoms of the group being nearly co-planar, the dihedral angle between CO_2_ and OCH_2_CH_3_ moieties being 7.95 (14)°; the other ethoxycarbonyl chain (O4—C21—O5—C22—C23) is also s-*cis*, the ethyl and the carboxylate moieties in a *gauche* relationship, the torsion angle of C21—O5—C22—C23 being 85.33 (9)°. The geometry of the title compound is similar to that of pirrolidinones (Nigam *et al.* 1989), (Ray *et al.*, 2004), (Ray *et al.*, 2010), (Kandasamy *et al.*, 1995). The crystal structure contains van der Waals and C—H···O weak interactions, the latter are listed in Table 1. Carbonyl O atoms O1, O2 and O4 interact with two H atoms; such intermolecular interactions could be classified as supportive (Desiraju, 2005). Inversion dimers are formed involving oxygen atoms O2 and O4; in the first case, the same pair of molecules are linked, each oxygen O2 of one molecule interacting with H atoms H2a and H13 of the other (symmetry code: -*x*, 1 - *y*, 2 - *z*). When oxygen O4 is considered, three molecules participate; there is an inversion dimer due to the intermolecular interactions between oxygen O4 and hydrogen H2b (symmetry code: -*x*, 1 - *y*, 1 - *z*), and the same applies for O4 and H22*b* (symmetry code: 1 - *x*, 1 - *y*, 1 - *z*). In addition to those dimers, the interaction of oxygen O1 with H atoms H15 (symmetry code: *x*, 1 + *y*, *z*) and H22*a* (symmetry code: -1 + *x*, *y*, *z*) results in sheets propagating in the *ab* plane. The angle between the two C—H..O hydrogen bonds bifurcated at O1 (C15—H15—O1 and C22—H22*a*—O1) is almost a right angle (86.7°).

### Experimental

The title compound was synthesized *via* an intermolecular Michael addition reaction, followed by an intramolecular amidification reaction, between diethyl 4-methylanilinomalonate (synthesized by the condensation reaction between 4-methylaniline and diethyl bromomalonate) in the presence of triethylamine, using dry benzene as solvent. Single crystals were grown by slow evaporation at room temperature of a solution of the resulting compound in 2-propanol. Yield 79%. Colourless solid [m.p. 401–402 K (ethyl acetate-petroleum ether)]; ν_max_(liquid film)/cm^-1^ 1727.75, 1638.76; δH (200 MHz; CDCl_3_; Me_4_Si) 0.79 (3*H*, t, J 7.04, OCH_2_CH_3_), 0.94 (3*H*, t, J 7.24, OCH_2_CH_3_), 2.34 (3*H*, s, ArCH_3_), 2.98–3.05 (2*H*, dd, J 5.2 and J 9.15, NCOCH_2_), 3.47–3.56 (1*H*, m, OCH_2_CH_3_), 3.81–4.16 (3*H*, m, OCH_2_CH_3_), 4.6 (1*H*, t, J 9.44, C(3)HPh), 7.19–7.25 (4*H*, m, ArH), 7.3–7.37 (5*H*, m, ArH). δC (100 MHz; CDCl_3_; Me_4_Si) 13.36, 13.47 (2× OCH_2_CH_3_), 21.18 (ArCH_3_), 35.12 (C(4)H_2_), 45.21 (C(3)HPh), 61.80, 62.28 (2×OCH_2_CH_3_), 79.34 (C(2)), 128.12 (C_P_), 128.45(2C_b_), 128.49 (2C_n_), 128.54 (2C_o_), 129.62 (2C_c_), 134.17 (C_d_), 136.65 (C_a_), 138.29 (C_m_), 167.06, 167.31 (2× COOCH_2_CH_3_), 174.90 (NCO).

### Refinement

Hydrogen atoms were found in subsequent difference Fourier maps and included in observed positions and refined as free isotropic atoms.

ALERTs all level C PLAT029_ALERT_3_C _diffrn_measured_fraction_theta_full Low. 0.98 RESPONSE: REason unknown. Optimized strategy by the software in order to get high completeness to resolution=0.75 A and enough redundancy and cut off in the refinement at 2theta=51, optimizing the the ratio parameters/data.

PLAT153_ALERT_1_C The su's on the Cell Axes are Equal (*x* 100000) 20 A ng. RESPONSE: It is not a mistake.

PLAT154_ALERT_1_G The su's on the Cell Angles are Equal (*x* 10000) 100 Deg. RESPONSE: It is not a mistake.

PLAT793_ALERT_4_G The Model has Chirality at C3 (Verify) ···. *R* RESPONSE: The compound is in a racemic mixture.

### Crystal data

**Table:**

C_23_H_25_NO_5_	*Z* = 2
*M**_r_* = 395.44	*F*(000) = 420
Triclinic, *P*1	*D*_x_ = 1.297 Mg m^−^^3^
Hall symbol: -P 1	Melting point: 401 K
*a* = 9.4905 (2) Å	Mo *K*α radiation, λ = 0.71073 Å
*b* = 10.6167 (2) Å	Cell parameters from 9079 reflections
*c* = 10.8198 (2) Å	θ = 2.5–28.2°
α = 93.014 (1)°	µ = 0.09 mm^−^^1^
β = 95.167 (1)°	*T* = 100 K
γ = 110.537 (1)°	Block, colourless
*V* = 1012.60 (3) Å^3^	0.42 × 0.30 × 0.12 mm

### Data collection

**Table:**

Bruker APEXII area-detector diffractometer	3672 independent reflections
Radiation source: fine-focus sealed tube	3331 reflections with *I* > 2σ(*I*)
graphite	*R*_int_ = 0.020
phi and ω scans	θ_max_ = 25.5°, θ_min_ = 2.5°
Absorption correction: multi-scan (*SADABS*; Bruker, 2009)	*h* = −11→11
*T*_min_ = 0.964, *T*_max_ = 0.996	*k* = −12→12
14991 measured reflections	*l* = −13→13

### Refinement

**Table:**

Refinement on *F*^2^	Primary atom site location: structure-invariant direct methods
Least-squares matrix: full	Secondary atom site location: difference Fourier map
*R*[*F*^2^ > 2σ(*F*^2^)] = 0.031	Hydrogen site location: inferred from neighbouring sites
*wR*(*F*^2^) = 0.091	All H-atom parameters refined
*S* = 1.87	*w* = 1/[σ^2^(*F*_o_^2^) + (0.0352*P*)^2^] where *P* = (*F*_o_^2^ + 2*F*_c_^2^)/3
3672 reflections	(Δ/σ)_max_ = 0.001
362 parameters	Δρ_max_ = 0.22 e Å^−^^3^
0 restraints	Δρ_min_ = −0.22 e Å^−^^3^

### Special details

**Table:**

Experimental. Data was collected using a X8 *APEX* II BRUKER-Nonius diffractometer equipped with an KRYOFLEX low-temperature apparatus operating at 100 K. A suitable crystal was chosen and mounted on a glass fiber using grease. Data were measured using omega scans of 0.5° per frame for 10 s, such that a total of 2870 frames were collected in a optimized strategy and with a final resolution of 0.75 Å. Data integration and reduction was performed using the *APEX2* (Bruker, 2009) software suite. Absorption corrections were applied using *SADABS* (Bruker, 2009). The structures are solved by direct methods using the *SHELX97* program and refined by least squares on F^2^*SHELXL97*, incorporated in the Apex2 software suite.
Geometry. All e.s.d.'s (except the e.s.d. in the dihedral angle between two l.s. planes) are estimated using the full covariance matrix. The cell e.s.d.'s are taken into account individually in the estimation of e.s.d.'s in distances, angles and torsion angles; correlations between e.s.d.'s in cell parameters are only used when they are defined by crystal symmetry. An approximate (isotropic) treatment of cell e.s.d.'s is used for estimating e.s.d.'s involving l.s. planes.
Refinement. Refinement of *F*^2^ against ALL reflections. The weighted *R*-factor *wR* and goodness of fit *S* are based on *F*^2^, conventional *R*-factors *R* are based on *F*, with *F* set to zero for negative *F*^2^. The threshold expression of *F*^2^ > σ(*F*^2^) is used only for calculating *R*-factors(gt) *etc*. and is not relevant to the choice of reflections for refinement. *R*-factors based on *F*^2^ are statistically about twice as large as those based on *F*, and *R*- factors based on ALL data will be even larger.All non-hydrogen atoms were refined anisotropically. Hydrogen were found in subsequent difference Fourier maps and included as isotropic atoms.

### Fractional atomic coordinates and isotropic or equivalent isotropic displacement parameters (Å^2^)

**Table:**

	*x*	*y*	*z*	*U*_iso_*/*U*_eq_	
O1	−0.06859 (8)	0.71231 (8)	0.72064 (8)	0.0250 (2)	
O2	0.19397 (9)	0.56920 (8)	0.97081 (7)	0.0205 (2)	
O3	0.28043 (8)	0.41475 (7)	0.88942 (6)	0.01674 (18)	
O4	0.27852 (9)	0.49468 (9)	0.55611 (7)	0.0247 (2)	
O5	0.43697 (8)	0.59245 (8)	0.72998 (6)	0.01664 (18)	
N1	0.13851 (9)	0.64837 (9)	0.73404 (8)	0.0144 (2)	
C1	−0.01411 (11)	0.62475 (11)	0.72555 (9)	0.0162 (2)	
C2	−0.09624 (12)	0.47497 (11)	0.72503 (11)	0.0169 (2)	
H2A	−0.1303 (14)	0.4554 (13)	0.8076 (13)	0.026 (3)*	
H2B	−0.1846 (14)	0.4437 (13)	0.6609 (12)	0.022 (3)*	
C3	0.02044 (11)	0.41224 (11)	0.69452 (10)	0.0150 (2)	
H3	0.0252 (13)	0.4114 (12)	0.6039 (12)	0.017 (3)*	
C4	0.17508 (11)	0.52637 (10)	0.74648 (9)	0.0140 (2)	
C5	0.24744 (11)	0.78151 (11)	0.73057 (9)	0.0146 (2)	
C6	0.34323 (13)	0.85116 (12)	0.83513 (10)	0.0215 (3)	
H6	0.3365 (15)	0.8085 (14)	0.9110 (14)	0.035 (4)*	
C7	0.44489 (13)	0.98064 (12)	0.82749 (11)	0.0244 (3)	
H7	0.5146 (15)	1.0322 (14)	0.8986 (13)	0.031 (3)*	
C8	0.45132 (12)	1.04361 (11)	0.71799 (10)	0.0204 (3)	
C9	0.35414 (12)	0.97144 (12)	0.61366 (10)	0.0218 (3)	
H9	0.3578 (15)	1.0138 (14)	0.5335 (13)	0.033 (4)*	
C10	0.25387 (12)	0.84142 (11)	0.61952 (10)	0.0196 (3)	
H10	0.1856 (16)	0.7897 (14)	0.5467 (13)	0.030 (3)*	
C11	0.55881 (16)	1.18611 (13)	0.71308 (13)	0.0290 (3)	
H11A	0.6210 (17)	1.2226 (15)	0.7902 (14)	0.037 (4)*	
H11B	0.5053 (18)	1.2474 (17)	0.6996 (15)	0.048 (4)*	
H11C	0.6186 (19)	1.1952 (16)	0.6448 (15)	0.048 (4)*	
C12	−0.00523 (11)	0.27271 (11)	0.73582 (10)	0.0163 (2)	
C13	−0.06002 (12)	0.23452 (11)	0.84876 (11)	0.0197 (2)	
H13	−0.0828 (14)	0.2983 (13)	0.9043 (12)	0.025 (3)*	
C14	−0.08302 (13)	0.10562 (12)	0.88368 (12)	0.0247 (3)	
H14	−0.1211 (16)	0.0818 (14)	0.9631 (13)	0.033 (4)*	
C15	−0.05170 (14)	0.01258 (12)	0.80671 (12)	0.0283 (3)	
H15	−0.0677 (15)	−0.0789 (15)	0.8318 (12)	0.033 (4)*	
C16	0.00369 (14)	0.04957 (13)	0.69484 (12)	0.0277 (3)	
H16	0.0251 (16)	−0.0136 (15)	0.6414 (13)	0.035 (4)*	
C17	0.02681 (13)	0.17826 (12)	0.65939 (11)	0.0216 (3)	
H17	0.0627 (15)	0.2023 (13)	0.5821 (13)	0.029 (3)*	
C18	0.21958 (11)	0.50969 (10)	0.88294 (9)	0.0140 (2)	
C19	0.30418 (13)	0.37111 (12)	1.01290 (10)	0.0198 (3)	
H19A	0.2082 (14)	0.3496 (12)	1.0498 (11)	0.019 (3)*	
H19B	0.3819 (13)	0.4486 (13)	1.0648 (11)	0.017 (3)*	
C20	0.35183 (15)	0.25173 (13)	0.99354 (12)	0.0266 (3)	
H20A	0.2751 (19)	0.1811 (17)	0.9407 (15)	0.045 (4)*	
H20B	0.3660 (15)	0.2159 (14)	1.0749 (13)	0.034 (4)*	
H20C	0.4514 (17)	0.2762 (14)	0.9584 (13)	0.037 (4)*	
C21	0.30183 (11)	0.53278 (11)	0.66516 (9)	0.0148 (2)	
C22	0.56966 (12)	0.61488 (13)	0.66233 (10)	0.0194 (3)	
H22A	0.6495 (14)	0.6216 (12)	0.7267 (11)	0.017 (3)*	
H22B	0.5482 (13)	0.5336 (13)	0.6052 (11)	0.018 (3)*	
C23	0.60496 (15)	0.74321 (15)	0.60006 (13)	0.0299 (3)	
H23A	0.6205 (15)	0.8196 (15)	0.6628 (13)	0.030 (4)*	
H23B	0.6949 (17)	0.7600 (15)	0.5572 (13)	0.038 (4)*	
H23C	0.5222 (17)	0.7335 (15)	0.5358 (14)	0.042 (4)*	

### Atomic displacement parameters (Å^2^)

**Table:**

	*U*^11^	*U*^22^	*U*^33^	*U*^12^	*U*^13^	*U*^23^
O1	0.0162 (4)	0.0157 (4)	0.0452 (5)	0.0083 (3)	0.0031 (3)	0.0041 (4)
O2	0.0276 (4)	0.0211 (4)	0.0159 (4)	0.0115 (3)	0.0066 (3)	0.0013 (3)
O3	0.0205 (4)	0.0187 (4)	0.0145 (4)	0.0108 (3)	0.0027 (3)	0.0044 (3)
O4	0.0195 (4)	0.0374 (5)	0.0148 (4)	0.0078 (4)	0.0034 (3)	−0.0028 (3)
O5	0.0119 (4)	0.0237 (4)	0.0151 (4)	0.0070 (3)	0.0034 (3)	0.0015 (3)
N1	0.0122 (4)	0.0123 (5)	0.0191 (5)	0.0048 (4)	0.0016 (3)	0.0026 (4)
C1	0.0141 (5)	0.0177 (6)	0.0177 (5)	0.0067 (4)	0.0021 (4)	0.0019 (4)
C2	0.0129 (5)	0.0153 (6)	0.0224 (6)	0.0048 (4)	0.0014 (4)	0.0027 (4)
C3	0.0144 (5)	0.0148 (5)	0.0150 (5)	0.0047 (4)	0.0013 (4)	0.0007 (4)
C4	0.0142 (5)	0.0139 (5)	0.0150 (5)	0.0063 (4)	0.0025 (4)	0.0008 (4)
C5	0.0122 (5)	0.0130 (5)	0.0200 (5)	0.0057 (4)	0.0033 (4)	0.0021 (4)
C6	0.0264 (6)	0.0183 (6)	0.0170 (6)	0.0048 (5)	0.0007 (5)	0.0038 (5)
C7	0.0267 (6)	0.0198 (6)	0.0200 (6)	0.0017 (5)	−0.0030 (5)	0.0003 (5)
C8	0.0178 (6)	0.0171 (6)	0.0253 (6)	0.0045 (5)	0.0037 (4)	0.0037 (5)
C9	0.0216 (6)	0.0214 (6)	0.0211 (6)	0.0052 (5)	0.0022 (5)	0.0072 (5)
C10	0.0177 (6)	0.0191 (6)	0.0189 (6)	0.0036 (5)	−0.0017 (4)	0.0022 (5)
C11	0.0287 (7)	0.0215 (7)	0.0289 (7)	−0.0006 (6)	0.0007 (6)	0.0051 (5)
C12	0.0122 (5)	0.0143 (6)	0.0210 (5)	0.0036 (4)	−0.0006 (4)	0.0002 (4)
C13	0.0186 (6)	0.0172 (6)	0.0241 (6)	0.0070 (5)	0.0035 (4)	0.0020 (5)
C14	0.0234 (6)	0.0208 (6)	0.0300 (7)	0.0070 (5)	0.0043 (5)	0.0079 (5)
C15	0.0283 (7)	0.0149 (6)	0.0410 (7)	0.0080 (5)	−0.0018 (5)	0.0044 (5)
C16	0.0297 (7)	0.0191 (6)	0.0353 (7)	0.0125 (5)	−0.0013 (5)	−0.0066 (5)
C17	0.0206 (6)	0.0216 (6)	0.0228 (6)	0.0088 (5)	0.0011 (5)	−0.0024 (5)
C18	0.0112 (5)	0.0135 (5)	0.0169 (5)	0.0030 (4)	0.0038 (4)	0.0026 (4)
C19	0.0224 (6)	0.0224 (6)	0.0147 (5)	0.0077 (5)	0.0005 (5)	0.0058 (5)
C20	0.0312 (7)	0.0267 (7)	0.0259 (7)	0.0152 (6)	0.0003 (5)	0.0081 (5)
C21	0.0156 (5)	0.0148 (5)	0.0156 (5)	0.0070 (4)	0.0022 (4)	0.0032 (4)
C22	0.0128 (5)	0.0298 (7)	0.0181 (6)	0.0094 (5)	0.0058 (4)	0.0041 (5)
C23	0.0211 (6)	0.0374 (8)	0.0338 (7)	0.0104 (6)	0.0099 (6)	0.0143 (6)

### Geometric parameters (Å, °)

**Table:**

O1—C1	1.2132 (13)	C9—H9	0.996 (14)
O2—C18	1.2035 (12)	C10—H10	0.977 (15)
O3—C18	1.3278 (12)	C11—H11A	0.955 (16)
O3—C19	1.4611 (12)	C11—H11B	0.965 (17)
O4—C21	1.2019 (13)	C11—H11C	0.960 (17)
O5—C21	1.3264 (13)	C12—C13	1.3952 (15)
O5—C22	1.4671 (12)	C12—C17	1.3978 (16)
N1—C1	1.3741 (13)	C13—C14	1.3867 (16)
N1—C5	1.4337 (13)	C13—H13	0.974 (13)
N1—C4	1.4633 (13)	C14—C15	1.3862 (18)
C1—C2	1.5049 (15)	C14—H14	0.974 (14)
C2—C3	1.5294 (14)	C15—C16	1.3840 (18)
C2—H2A	0.985 (13)	C15—H15	0.986 (14)
C2—H2B	0.985 (13)	C16—C17	1.3862 (17)
C3—C12	1.5131 (15)	C16—H16	0.949 (15)
C3—C4	1.5716 (14)	C17—H17	0.945 (14)
C3—H3	0.985 (12)	C19—C20	1.4987 (17)
C4—C18	1.5353 (14)	C19—H19A	0.985 (13)
C4—C21	1.5380 (14)	C19—H19B	0.994 (13)
C5—C6	1.3813 (15)	C20—H20A	0.957 (17)
C5—C10	1.3851 (15)	C20—H20B	0.994 (14)
C6—C7	1.3867 (16)	C20—H20C	1.004 (15)
C6—H6	0.955 (15)	C22—C23	1.4964 (17)
C7—C8	1.3865 (16)	C22—H22A	0.963 (12)
C7—H7	0.969 (14)	C22—H22B	0.983 (13)
C8—C9	1.3920 (16)	C23—H23A	0.990 (15)
C8—C11	1.5059 (16)	C23—H23B	0.974 (15)
C9—C10	1.3833 (16)	C23—H23C	0.974 (16)
			
C18—O3—C19	116.52 (8)	H11B—C11—H11C	104.4 (13)
C21—O5—C22	117.09 (8)	C13—C12—C17	118.39 (10)
C1—N1—C5	121.50 (9)	C13—C12—C3	122.07 (9)
C1—N1—C4	113.50 (8)	C17—C12—C3	119.54 (10)
C5—N1—C4	125.00 (8)	C14—C13—C12	120.68 (11)
O1—C1—N1	124.33 (10)	C14—C13—H13	119.0 (8)
O1—C1—C2	127.73 (9)	C12—C13—H13	120.3 (8)
N1—C1—C2	107.93 (9)	C15—C14—C13	120.39 (12)
C1—C2—C3	104.67 (8)	C15—C14—H14	120.7 (8)
C1—C2—H2A	108.8 (8)	C13—C14—H14	118.9 (8)
C3—C2—H2A	112.7 (7)	C16—C15—C14	119.46 (11)
C1—C2—H2B	110.5 (8)	C16—C15—H15	120.2 (8)
C3—C2—H2B	110.7 (7)	C14—C15—H15	120.3 (8)
H2A—C2—H2B	109.5 (10)	C15—C16—C17	120.41 (11)
C12—C3—C2	116.32 (9)	C15—C16—H16	120.0 (9)
C12—C3—C4	116.64 (8)	C17—C16—H16	119.6 (9)
C2—C3—C4	102.81 (8)	C16—C17—C12	120.67 (11)
C12—C3—H3	110.1 (7)	C16—C17—H17	120.0 (8)
C2—C3—H3	107.7 (7)	C12—C17—H17	119.3 (8)
C4—C3—H3	102.0 (7)	O2—C18—O3	125.34 (9)
N1—C4—C18	111.92 (8)	O2—C18—C4	124.12 (9)
N1—C4—C21	108.16 (8)	O3—C18—C4	110.40 (8)
C18—C4—C21	111.84 (8)	O3—C19—C20	106.36 (9)
N1—C4—C3	101.81 (8)	O3—C19—H19A	107.5 (7)
C18—C4—C3	110.42 (8)	C20—C19—H19A	113.3 (7)
C21—C4—C3	112.27 (8)	O3—C19—H19B	107.9 (7)
C6—C5—C10	119.81 (10)	C20—C19—H19B	113.4 (7)
C6—C5—N1	121.64 (9)	H19A—C19—H19B	108.1 (10)
C10—C5—N1	118.54 (9)	C19—C20—H20A	110.4 (9)
C5—C6—C7	119.44 (10)	C19—C20—H20B	109.6 (8)
C5—C6—H6	118.6 (9)	H20A—C20—H20B	107.7 (12)
C7—C6—H6	122.0 (9)	C19—C20—H20C	112.2 (8)
C8—C7—C6	121.76 (11)	H20A—C20—H20C	110.3 (12)
C8—C7—H7	116.5 (8)	H20B—C20—H20C	106.5 (11)
C6—C7—H7	121.8 (8)	O4—C21—O5	125.77 (10)
C7—C8—C9	117.84 (10)	O4—C21—C4	123.35 (9)
C7—C8—C11	120.84 (10)	O5—C21—C4	110.77 (8)
C9—C8—C11	121.31 (10)	O5—C22—C23	110.51 (9)
C10—C9—C8	120.96 (10)	O5—C22—H22A	103.6 (7)
C10—C9—H9	119.6 (8)	C23—C22—H22A	110.8 (7)
C8—C9—H9	119.4 (8)	O5—C22—H22B	106.9 (7)
C9—C10—C5	120.17 (10)	C23—C22—H22B	114.4 (7)
C9—C10—H10	121.6 (8)	H22A—C22—H22B	109.9 (10)
C5—C10—H10	118.2 (8)	C22—C23—H23A	109.7 (8)
C8—C11—H11A	112.6 (9)	C22—C23—H23B	110.8 (9)
C8—C11—H11B	111.6 (10)	H23A—C23—H23B	110.0 (12)
H11A—C11—H11B	103.5 (13)	C22—C23—H23C	108.9 (9)
C8—C11—H11C	112.7 (10)	H23A—C23—H23C	111.5 (12)
H11A—C11—H11C	111.4 (13)	H23B—C23—H23C	106.0 (12)
			
C5—N1—C1—O1	−3.65 (16)	C6—C5—C10—C9	−1.04 (16)
C4—N1—C1—O1	176.36 (10)	N1—C5—C10—C9	177.92 (10)
C5—N1—C1—C2	176.93 (9)	C2—C3—C12—C13	−38.01 (14)
C4—N1—C1—C2	−3.06 (11)	C4—C3—C12—C13	83.65 (12)
O1—C1—C2—C3	163.95 (11)	C2—C3—C12—C17	142.13 (10)
N1—C1—C2—C3	−16.65 (11)	C4—C3—C12—C17	−96.21 (12)
C1—C2—C3—C12	156.77 (9)	C17—C12—C13—C14	−0.44 (16)
C1—C2—C3—C4	28.05 (10)	C3—C12—C13—C14	179.69 (10)
C1—N1—C4—C18	−97.26 (10)	C12—C13—C14—C15	0.05 (17)
C5—N1—C4—C18	82.75 (11)	C13—C14—C15—C16	0.39 (18)
C1—N1—C4—C21	139.09 (9)	C14—C15—C16—C17	−0.44 (18)
C5—N1—C4—C21	−40.89 (12)	C15—C16—C17—C12	0.04 (18)
C1—N1—C4—C3	20.66 (10)	C13—C12—C17—C16	0.40 (16)
C5—N1—C4—C3	−159.32 (9)	C3—C12—C17—C16	−179.73 (10)
C12—C3—C4—N1	−157.57 (8)	C19—O3—C18—O2	6.15 (14)
C2—C3—C4—N1	−29.05 (10)	C19—O3—C18—C4	−169.70 (8)
C12—C3—C4—C18	−38.57 (12)	N1—C4—C18—O2	16.45 (14)
C2—C3—C4—C18	89.95 (9)	C21—C4—C18—O2	138.00 (10)
C12—C3—C4—C21	86.98 (11)	C3—C4—C18—O2	−96.21 (12)
C2—C3—C4—C21	−144.51 (8)	N1—C4—C18—O3	−167.65 (8)
C1—N1—C5—C6	109.35 (12)	C21—C4—C18—O3	−46.09 (11)
C4—N1—C5—C6	−70.67 (14)	C3—C4—C18—O3	79.70 (10)
C1—N1—C5—C10	−69.59 (13)	C18—O3—C19—C20	172.04 (9)
C4—N1—C5—C10	110.40 (11)	C22—O5—C21—O4	0.53 (16)
C10—C5—C6—C7	0.02 (16)	C22—O5—C21—C4	−175.69 (8)
N1—C5—C6—C7	−178.91 (10)	N1—C4—C21—O4	−85.80 (12)
C5—C6—C7—C8	1.25 (18)	C18—C4—C21—O4	150.51 (10)
C6—C7—C8—C9	−1.45 (17)	C3—C4—C21—O4	25.74 (14)
C6—C7—C8—C11	177.81 (12)	N1—C4—C21—O5	90.53 (10)
C7—C8—C9—C10	0.40 (17)	C18—C4—C21—O5	−33.16 (12)
C11—C8—C9—C10	−178.86 (11)	C3—C4—C21—O5	−157.93 (8)
C8—C9—C10—C5	0.83 (17)	C21—O5—C22—C23	84.62 (12)

### Hydrogen-bond geometry (Å, °)

**Table:**

*D*—H···*A*	*D*—H	H···*A*	*D*···*A*	*D*—H···*A*
C2—H2A···O2^i^	0.985 (14)	2.529 (14)	3.5096 (14)	173.4 (10)
C3—H3···O4	0.984 (13)	2.369 (13)	2.8814 (14)	111.7 (9)
C6—H6···O2	0.955 (15)	2.573 (14)	3.3143 (14)	134.7 (11)
C13—H13···O2^i^	0.975 (14)	2.453 (14)	3.4128 (14)	168.3 (12)
C15—H15···O1^ii^	0.987 (15)	2.462 (15)	3.2184 (15)	133.2 (10)
C22—H22A···O1^iii^	0.963 (13)	2.513 (14)	3.2100 (15)	129.2 (9)
C22—H22B···O4^iv^	0.983 (13)	2.579 (13)	3.2426 (14)	124.9 (10)

Symmetry codes: (i) −*x*, −*y*+1, −*z*+2; (ii) *x*, *y*−1, *z*; (iii) *x*+1, *y*, *z*; (iv) −*x*+1, −*y*+1, −*z*+1.
